# Supplementary material for: The finite state projection based Fisher information matrix approach to estimate information and optimize single-cell experiments
Source: PLoS Comput Biol. 2019 Jan 15;15(1):e1006365. doi: 10.1371/journal.pcbi.1006365 (PMC6355035; doi:10.1371/journal.pcbi.1006365)
Supplement: S1 Fig — (PDF) [file pcbi.1006365.s002.pdf]

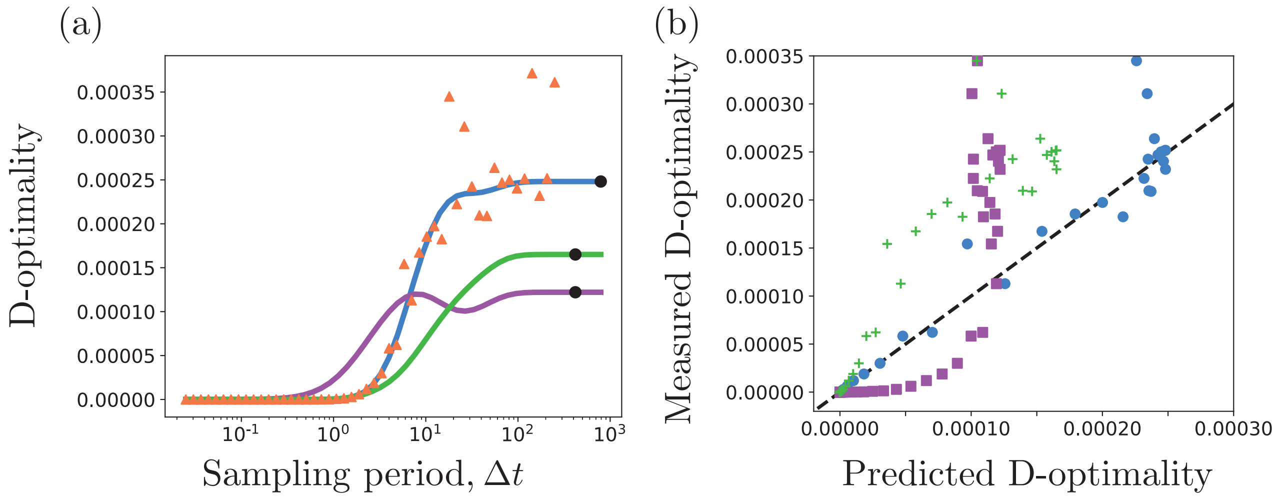

Figure S1: Optimal experiment design for the bursting gene expression model using the determinant of the FIM, D-optimality. (a) The D-optimality criteria for the FSP-FIM (blue), LNA-FIM (purple) and SM-FIM (green) for different sampling periods Δt. Orange triangles represent the D-optimality confirmed using 200 simulated data sets for each potential sampling period. Optimal sampling periods are given by black circles. (b) Comparison of the FSP-FIM at the the reference parameter set (x-axis) and the observed information (y-axis) for various sampling periods using the FSP-FIM (blue circles), LNA-FIM (purple squares), and SM-FIM (green crosses). Kinetic parameters are $k_{on} = 0.05$ min^{-1}, $k_{off} = 0.15$ min^{-1}, $k_r = 5$ molecules/min, and $\gamma = 0.05$ min^{-1}.
