# Supplementary material for: The finite state projection based Fisher information matrix approach to estimate information and optimize single-cell experiments
Source: PLoS Comput Biol. 2019 Jan 15;15(1):e1006365. doi: 10.1371/journal.pcbi.1006365 (PMC6355035; doi:10.1371/journal.pcbi.1006365)
Supplement: S2 Fig — (PDF) [file pcbi.1006365.s003.pdf]

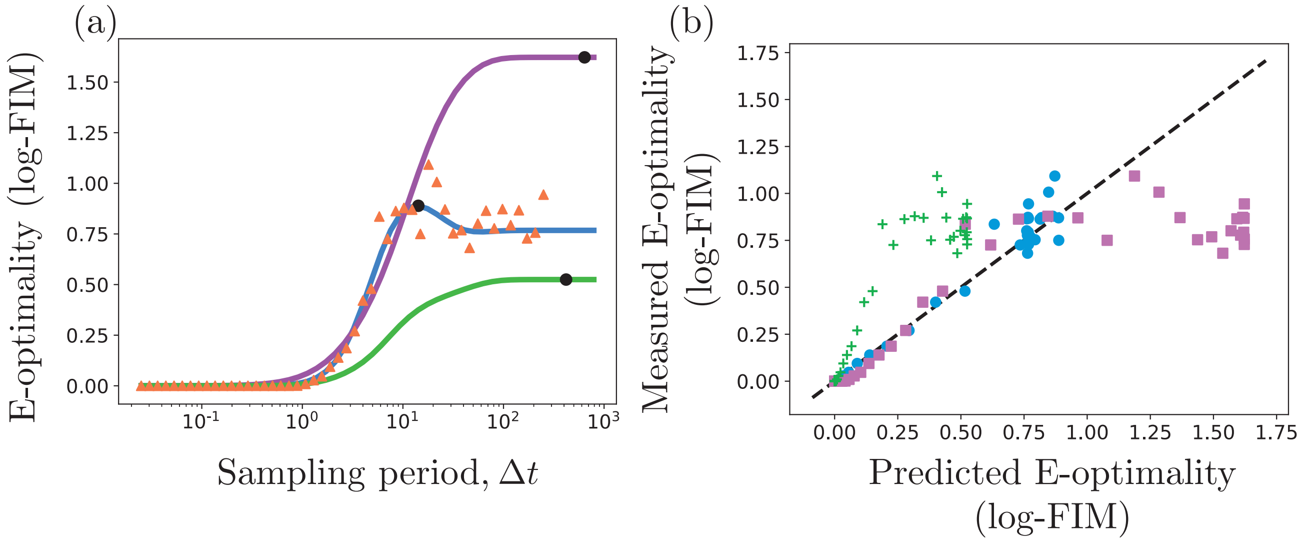

Figure S2: Optimal experiment design for the bursting gene expression model using E-optimality determined using the logarithmic parameterization of the FIM. The FSP-FIM (blue), LNA-FIM (purple) and SM-FIM (green) are shown for different sampling periods Δt. Orange triangles represent the E-optimality confirmed using 200 simulated data sets for each potential sampling period, where the optimal sampling periods are given by black circles.
