# Supplementary material for: The finite state projection based Fisher information matrix approach to estimate information and optimize single-cell experiments
Source: PLoS Comput Biol. 2019 Jan 15;15(1):e1006365. doi: 10.1371/journal.pcbi.1006365 (PMC6355035; doi:10.1371/journal.pcbi.1006365)
Supplement: S3 Fig — (PDF) [file pcbi.1006365.s004.pdf]

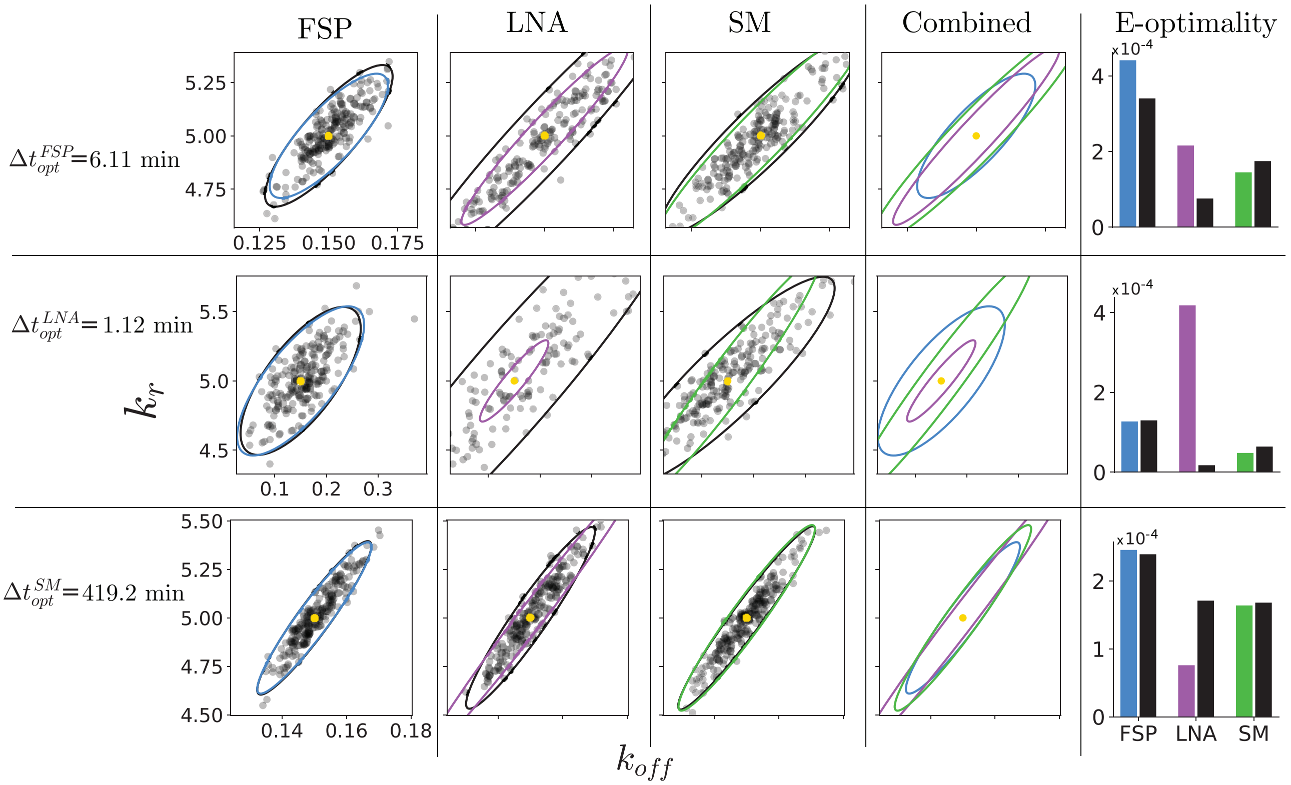

Figure S3: Verification of the optimal experiment design for the sampling period Δt of a bursting gene expression model. For each optimal sampling period, we simulated 200 data sets and find the MLE estimates for each data set (black circles) using each likelihood function (FSP, LNA and sample moments). The top row uses a Δt according to the optimal sampling period found using the FSP-FIM, the middle row uses the optimal Δt found using the LNA-FIM, and the bottom row using the SM-FIM, using the regular parameterization of the FIM (i.e. the black circles in Fig. 5).
