# Supplementary material for: The finite state projection based Fisher information matrix approach to estimate information and optimize single-cell experiments
Source: PLoS Comput Biol. 2019 Jan 15;15(1):e1006365. doi: 10.1371/journal.pcbi.1006365 (PMC6355035; doi:10.1371/journal.pcbi.1006365)
Supplement: S4 Fig — (PDF) [file pcbi.1006365.s005.pdf]

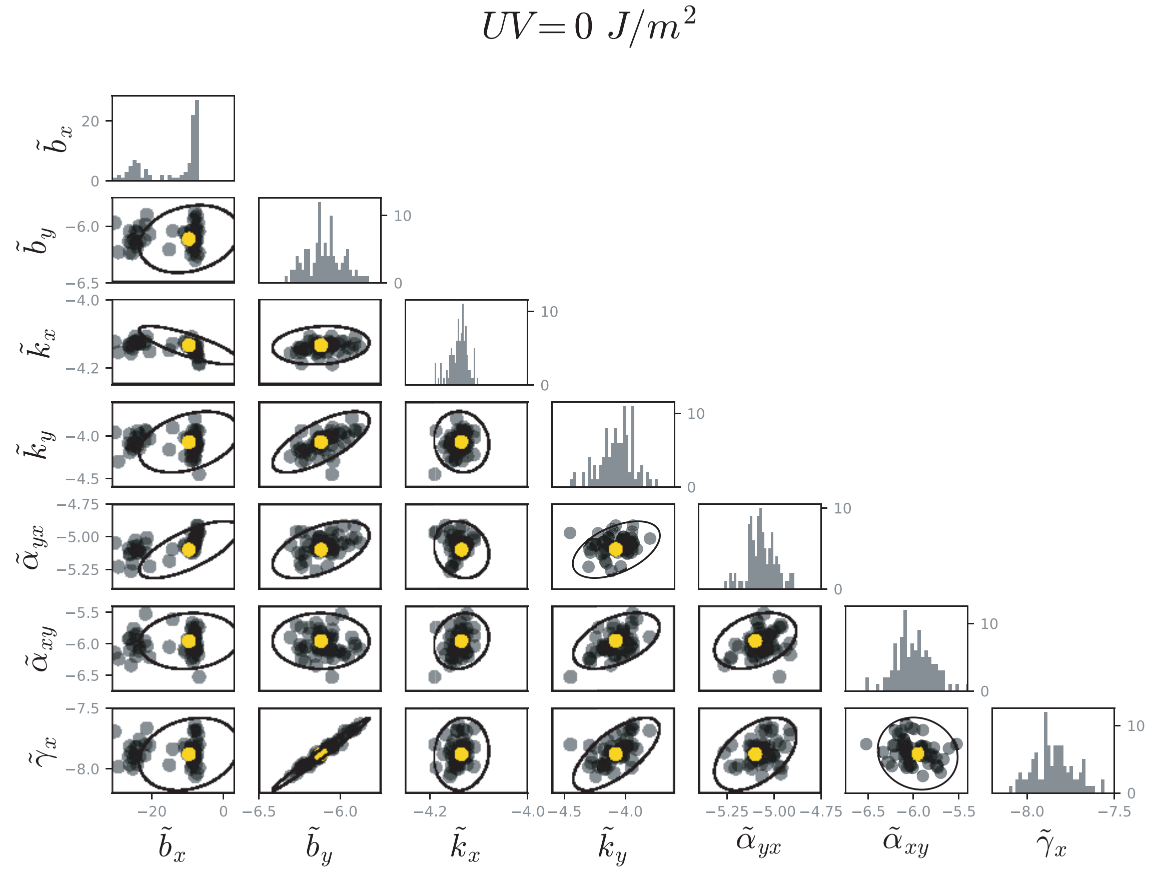

Figure S4: Verification of the FSP-FIM for the seven free parameters for the toggle model. Each black circle corresponds to the logarithm of an MLE estimate, $\log \hat{\boldsymbol{\theta}}$ for 100 different simulated data sets. The gold circle corresponds to the reference parameter set, $\log \boldsymbol{\theta}^*$. The 95% ellipse corresponding to the log-FSP-FIM is shown in black. The tilde corresponds to the log of each parameter, i.e. $\tilde{b}_x = \log b_x$.
