# Supplementary material for: The finite state projection based Fisher information matrix approach to estimate information and optimize single-cell experiments
Source: PLoS Comput Biol. 2019 Jan 15;15(1):e1006365. doi: 10.1371/journal.pcbi.1006365 (PMC6355035; doi:10.1371/journal.pcbi.1006365)
Supplement: S5 Fig — (PDF) [file pcbi.1006365.s006.pdf]

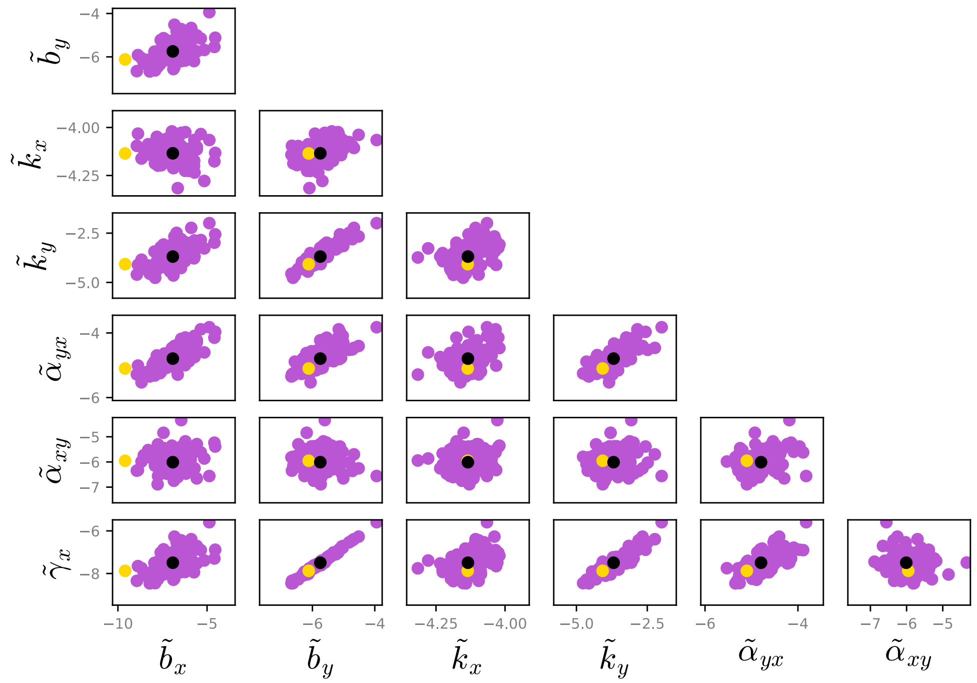

Figure S5: Parameters were sampled 100 times from a log-normal distribution evaluated about the reference parameter set $\hat{\boldsymbol{\theta}}_0$, shown in black. The covariance of this distribution was chosen according to the inverse of the FIM evaluated for an experiment with 0 UV, $t = [1, 4, 8]$ hr, and 100 measurements at each time point. For reference, the gold parameters are the 'true' parameters for the model. The tilde corresponds to the log of each parameter, i.e. $\tilde{b}_x = \log b_x$.
