# Supplementary material for: The finite state projection based Fisher information matrix approach to estimate information and optimize single-cell experiments
Source: PLoS Comput Biol. 2019 Jan 15;15(1):e1006365. doi: 10.1371/journal.pcbi.1006365 (PMC6355035; doi:10.1371/journal.pcbi.1006365)
Supplement: S6 Fig — (PDF) [file pcbi.1006365.s007.pdf]

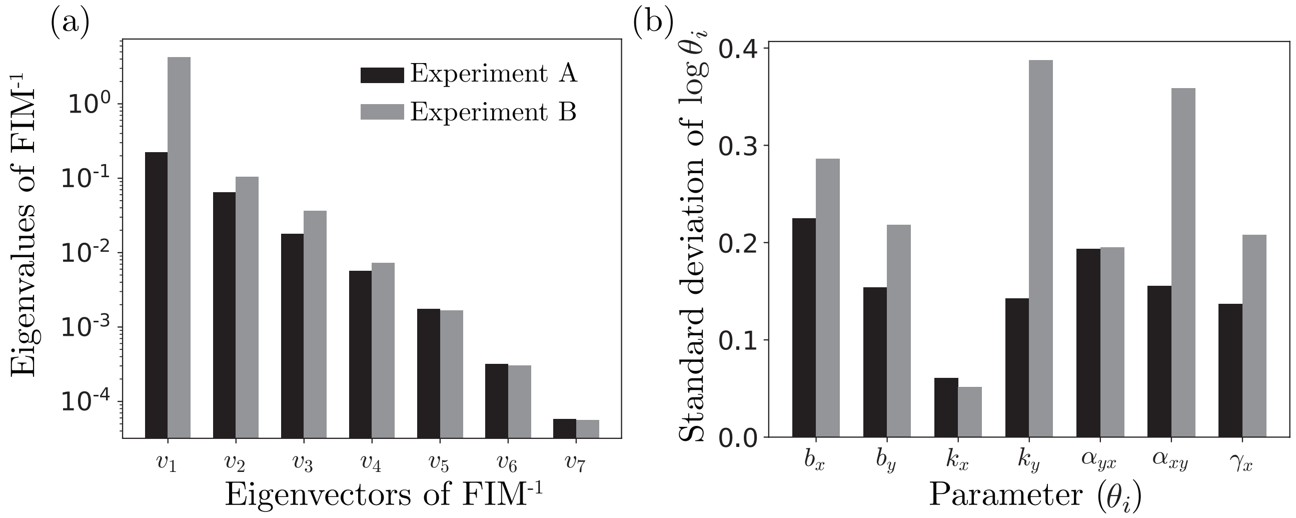

Figure S6: (a) The eigenvalues of the inverse of the Fisher information (i.e. the CRB) for the two experiments in Fig. 7(c) in the main text. Note that the y-axis is on a logarithmic scale. Lower values correspond to lower parameter uncertainty. (b) The effect of Experiment A and B on standard deviations of $\log \theta_i$.
