# Supplementary material for: The finite state projection based Fisher information matrix approach to estimate information and optimize single-cell experiments
Source: PLoS Comput Biol. 2019 Jan 15;15(1):e1006365. doi: 10.1371/journal.pcbi.1006365 (PMC6355035; doi:10.1371/journal.pcbi.1006365)
Supplement: S7 Fig — (PDF) [file pcbi.1006365.s008.pdf]

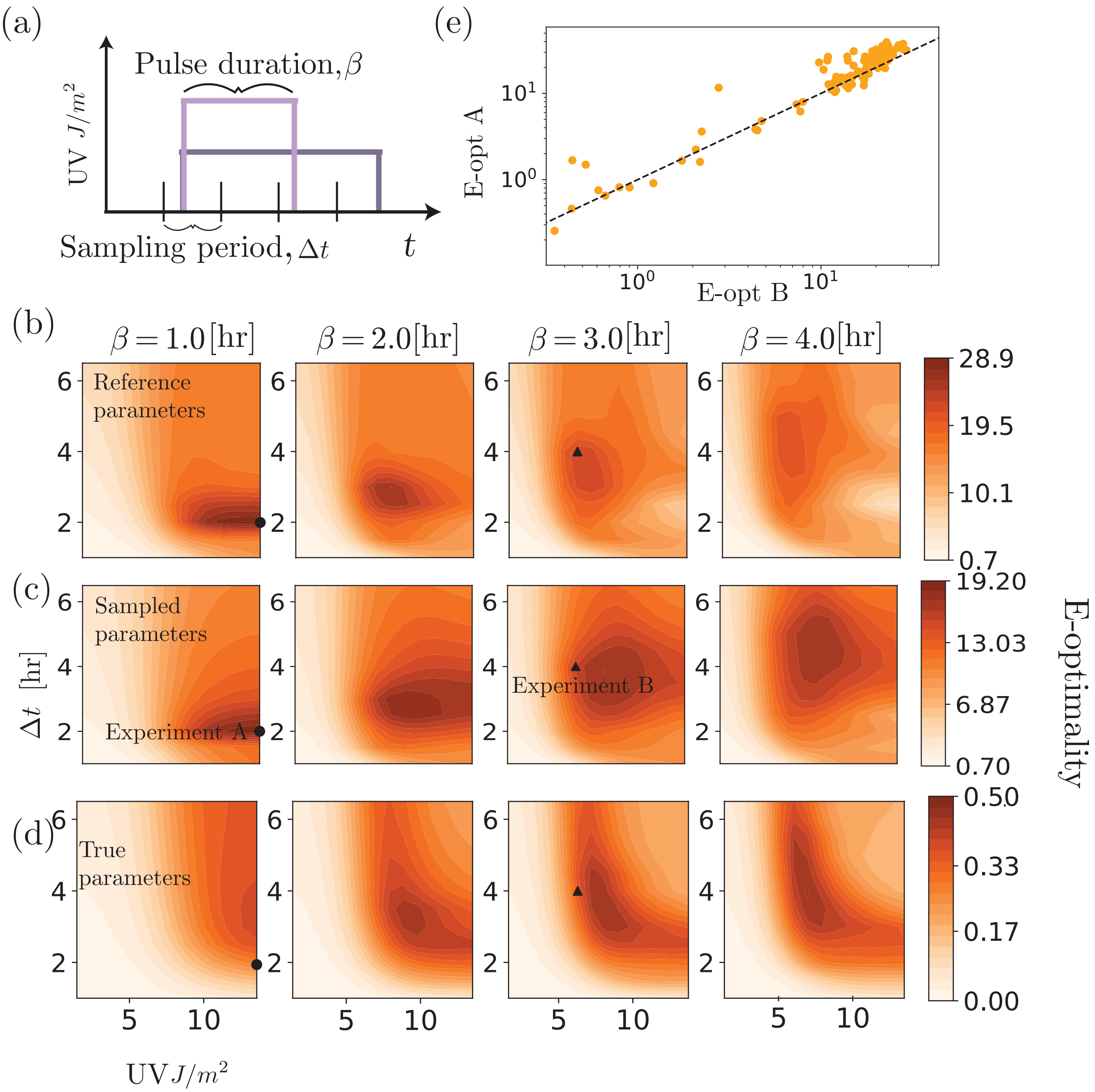

Figure S7: Toggle model experiment design with non-zero initial sensitivities (a) Degradation rate of λcI is controlled by UV as shown in Fig. 7(a). The magnitude and duration (β) of UV exposure are free experiment design parameters, along with the time between measurements Δt. (b) E-optimality (the smallest eigenvalue of the FIM) versus the 3-dimensional experiment design space, where the FIM is computed using (b) the reference parameter set, (c) by averaging the E-optimality over 100 unique parameter sets and (d) using the "true" parameter values. The black circle is the optimal design chosen according to (c). The black triangle denotes a nearby, but less informative, experiment. (e) For the experiments corresponding to the black circle and triangle in (b-d), E-optimality values are shown for each sampled parameter set.
