# Supplementary material for: The finite state projection based Fisher information matrix approach to estimate information and optimize single-cell experiments
Source: PLoS Comput Biol. 2019 Jan 15;15(1):e1006365. doi: 10.1371/journal.pcbi.1006365 (PMC6355035; doi:10.1371/journal.pcbi.1006365)
Supplement: S1 Table — (PDF) [file pcbi.1006365.s009.pdf]

	$\log b_y$	$\log b_x$	$\log k_y$	$\log k_x$	$\log \alpha_{xy}$	$\log \alpha_{yx}$	$\log \gamma_x$
$\log b_y$	0.673	0.130	-0.017	0.194	0.165	0.039	0.135
$\log b_x$	0.130	0.182	0.010	0.177	0.082	-0.011	0.184
$\log k_y$	-0.017	0.010	0.004	0.007	0.007	0.006	0.009
$\log k_x$	0.194	0.176	0.007	0.212	0.093	0.041	0.177
$\log \alpha_{xy}$	0.165	0.082	0.007	0.093	0.084	0.038	0.08
$\log \alpha_{yx}$	0.038	-0.011	0.006	0.041	0.038	0.160	-0.023
$\log \gamma_x$	0.135	0.184	0.009	0.177	0.080	-0.023	0.187

Table 1: Variance and covariance of the log of each parameter for the toggle model for prior uncertainty in the toggle model. This covariance was chosen according to the inverse of the logarithmic parameterized FIM evaluated for an experiment with 0 UV, $t = [1, 4, 8]$ hr, and 100 measurements at each time point.
